# Evaluation of early manifestations of spreading pattern *Mycobacterium marinum* infection by ultrasonography

**DOI:** 10.1111/srt.13147

**Published:** 2022-04-03

**Authors:** Xuesong Wang, Qing Zhao, Jianke Li, Yongxia Liu, Shanshan Ma, Hong Liu, Jian Qin, Furen Zhang

**Affiliations:** ^1^ First Clinical Medical College Shandong University of Traditional Chinese Medicine Shandong China; ^2^ Shandong Provincial Hospital for Skin Diseases Shandong First Medical University Shandong China; ^3^ Shandong Provincial Institute of Dermatology and Venereology Shandong Academy of Medical Sciences Shandong China; ^4^ Radiological Department The Second Affiliated Hospital of Shandong First Medical University Shandong China

Dear Editor,

*Mycobacterium marinum* is a pathogenic mycobacterium found in skin and soft tissue infections and is associated with fish and bodies of water.^1^ *Mycobacterium marinum* infections can be divided into four types: a solitary papulonodular lesion, sporotrichoid form, deep infection, and disseminated infection. In about 25% of patients, the infection manifests in the sporotrichoid form. This type of infection is characterized by the distribution of subcutaneous nodules of lymphatic vessels that further develop into larger nodules or cysts if not treated in time.^2^

Ultrasound is a repeatable and radiation‐free detection method that can provide us with comprehensive imaging and real‐time guidance.^3^ Accurate and unique information about cutaneous and subcutaneous lesions can be transmitted to dermatologists through ultrasound technology, and we can use ultrasound to assess the lesions during the treatment of patients with sporotrichoid infections. As far as we know, an ultrasonic evaluation of mycobacterium infection nodules has not yet been reported.

**FIGURE 1:**
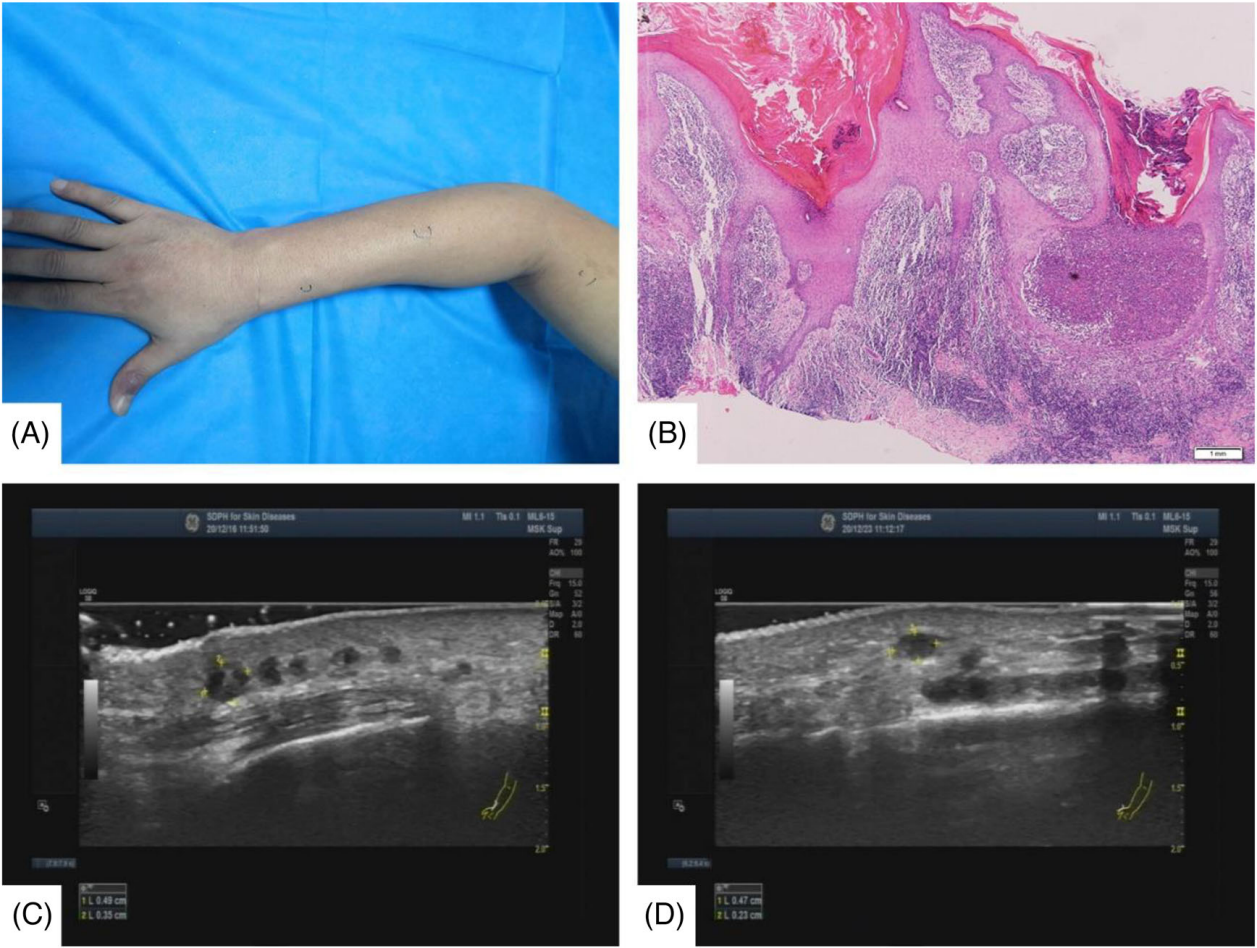
(A) Case 1: Lesions of patients at the first visit. (B) (Right thumb) Hyperkeratosis of the epidermis, incomplete keratosis, thickening of the spinous layer, and neutrophil abscess can be seen. Infiltration of lymphocytes, histiocytes, and plasma cells in the superficial dermis. PSA staining: No fungal hyphae and spores were found; acid‐fast staining: acid‐fast bacteria (–) (hematoxylin–eosin staining, ×40). (C) Ultrasonic images at first visit. (D) Ultrasound images at the second visit

As shown in Table 1, the infection occurred in the dominant hand of all three patients. Two of these patients had a clear history of fishbone injuries. As identified by the ultrasound, most of the nodules were subcutaneous, based on primary skin lesions in the early stage of the sporotrichoid form. Secondary spore‐like subcutaneous nodules could be seen disseminated in the affected upper limb, and the surrounding subcutaneous tissue was hypoechoic and beaded (Figure 1C). The average size of larger nodules was 0.61–0.41 cm patients came the first time, and it becomes to 0.6–0.35 cm the second time. These large nodules were scattered and distributed in subcutaneous tissue without invasion of the epidermis or dermis. The blood flow signal around the nodules was enhanced (Table 1).

**TABLE 1:** Clinical data of patients

	Case 1	Case 2	Case 3
Sex/Age	male/48	Female/59	Female /42
Course of disease	2 months	2 months	1 months
Clinical description	Red plaques, subcutaneous nodules	Red plaques, subcutaneous nodules	Red plaques, subcutaneous nodules
Lesions	Multiple	Multiple	Multiple
Size, cm (first time)	0.49 × 0.35	0.67 × 0.4	0.68 × 0.47
Size, cm (second time)	0.47 × 0.23	0.67 × 0.37	0.66 × 0.44
Site	Upper extremity, right hand	Upper extremity, right hand	Upper extremity, right hand
Mycobacterium marinum qPCR	Positive	Positive	Positive
Echogenicity	Hypoechoic subcutaneous nodular lesions with increased echogenicity of the neighboring subcutaneous	Hypoechoic nodules	Hypoechoic nodules
Extension	Dermis	Dermis	Dermis, subcutaneous
CDFI	Internal and peripheral	Internal and peripheral	Internal and peripheral
Boundary	Clear	Clear	Lack of clarity

Through ultrasound imagery, we can visually observe the spread of *M. marinum* in the lymphatics of these three patients. The number of subcutaneous nodules observed in the ultrasound is more than what can be seen on the surface (Figuer1A,C). This suggests that mycobacterial infections tend to be more severe than doctors observe, and we can only accurately assess the degree of an infection through the use of ultrasound. During treatment, we can directly observe the changes in the size and the number of lymph nodes using an ultrasound (Figure 1C,D). This can be directly applied to evaluate the clinical effect of our therapeutic regimen.

*Mycobacterium marinum* infections are usually treated with antibiotics such as rifampicin, clarithromycin, doxycycline, and ethambutol, among others. ^4^ However, there is no standard for the choice of treatment, and there is currently no clinical comparison among the various treatment regimens. Thus, the choice of treatment regimen depends largely on the personal experience of clinicians. ^5^ Ultrasound technology may be a feasible way to evaluate the efficacy of different treatments. Then nodules can be easily measured and these measurements used to help follow disease progression or response to therapy.
